# Gut Microbiota Composition Associated With *Clostridium difficile*-Positive Diarrhea and *C. difficile* Type in ICU Patients

**DOI:** 10.3389/fcimb.2020.00190

**Published:** 2020-05-11

**Authors:** Juping Duan, Xiujuan Meng, Sidi Liu, Pengcheng Zhou, Cui Zeng, Chenchao Fu, Qingya Dou, Anhua Wu, Chunhui Li

**Affiliations:** ^1^Infection Control Center, Xiangya Hospital, Central South University, Changsha, China; ^2^Changsha Hospital of Traditional Chinese Medicine, Changsha, China

**Keywords:** 16S rDNA sequencing, *Clostridium difficile*-associated diarrhea, gut microbiota, metagenome sequencing, *TcdA/TcdB*, *Clostridium difficile* infection, CDI, *Clostridium difficile*-positive diarrhea

## Abstract

The gut microbiota composition of intensive care unit (ICU) patients suffering from *Clostridium difficile*-positive diarrhea (CDpD) is poorly understood. This prospective study aims to use 16S rDNA (and metagenome) sequencing to compare the microbiota composition of 58 (and 5) ICU patients with CDpD (CDpD group), 33 (and 4) ICU patients with *C. difficile*-negative diarrhea (CDnD group), and 21 (and 5) healthy control subjects (control group), as well as CDpD patients in the A^+^B^+^ (*N* = 34; A/B: *C. difficile TcdA/B*), A^−^B^+^ (*N* = 7), and A^−^B^−^ (*N* = 17) subgroups. For 16S rDNA data, OTU clustering (tool: UPARSE), taxonomic assignment (tool: RDP classifier), α-diversity, and β-diversity analyses (tool: QIIME) were conducted. For metagenome data, metagenome assembly (tool: SOAPdenovo), gene calling (tools: MetaGeneMark, CD-HIT, and SoapAligner), unigene alignment (tool: DIAMOND), taxon difference analysis (tool: Metastats), and gene annotation (tool: DIAMOND) were performed. The microbial diversity of the CDpD group was lower than that of the CDnD and control groups. The abundances of 10 taxa (e.g., Deferribacteres, Cryptomycota, Acetothermia) were significantly higher in the CDpD group than in the CDnD group. The abundances of Saccharomycetes and Clostridia were significantly lower in CDpD in comparison with control. Some taxa were significantly different between the A^+^B^+^ and A^−^B^−^ subgroups. CDpD might relate to a decrease in beneficial taxa (i.e., Saccharomycetes and Clostridia) and an increase in harmful taxa (e.g., Deferribacteres, Cryptomycota, Acetothermia) in gut microbiota of ICU patients. *C. difficile* toxin type might be slightly associated with gut microbiota composition.

## Introduction

Antibiotics are often used in treating intensive care unit (ICU) patients (Vincent et al., 2016). However, antibiotic treatment is the most crucial risk factor associated *Clostridium difficile* (also known as *Peptoclostridium difficile*) infection (CDI) (Stevens et al., 2011), as antibiotics adversely affect the indigenous gut microbiota composition and decrease colonization resistance to *C. difficile* (Britton and Young, 2012). The clinical symptoms of CDI range from mild diarrhea to severe complications such as pseudomembranous colitis, toxic megacolon, bowel perforation, and death (Yassin et al., 2001; Rupnik et al., 2009; Shivashankar et al., 2013; Leffler and Lamont, 2015). The occurrence of CDI is 7.4~14.1 cases per 10,000 patient-days in ICU (Centers for Disease Control Prevention (CDC), 2012; Lee et al., 2015; Li et al., 2018).

Nevertheless, current antibiotic therapies for CDI such as vancomycin have limited efficacy (Bagdasarian et al., 2015), and fecal microbiota transplantation (FMT) is recommended as an alternative therapy in treating highly recurrent CDI that has failed to respond to vancomycin treatment (Kelly et al., 2015). The success of FMT emphasizes the importance of restoring the gut microbiome in CDI patients.

In recent years, high-throughput deep-sequencing of 16S rDNA and the metagenome has been applied to investigating microbiota composition. Milani et al. used 16S rDNA and metagenome sequencing to study the gut microbiota compositions of three groups of elderly (age ≥ 65) hospitalized patients, involving 30 CDI-negative patients not exposed to antibiotics, 29 CDI-negative patients exposed to antibiotics, and 25 CDI-positive patients (Milani et al., 2016). The microbial diversity of the CDI-positive group was significantly lower than that of the CDI-negative group. CDI was associated with the decrease in gut commensal bacteria such as *Bacteroides, Alistipes, Lachnospira*, and *Barnesiella*, while antibiotic treatment in CDI-negative patients might lead to the depletion of commensal bacteria such as *Alistipes* (Milani et al., 2016). Schubert et al. utilized 16S rDNA sequencing to study the gut microbiota compositions of CDI cases, diarrheal controls, and non-diarrheal controls (Schubert et al., 2014). Statistical models were developed for CDI and diarrhea by incorporating clinical and demographic data with microbiome data, and loss of several species in Ruminococcaceae, Lachnospiraceae, *Bacteroides* and Porphyromonadaceae might be associate with CDI (Schubert et al., 2014). However, the microbiome alterations related with diarrhea and *C. difficile*-positive diarrhea (CDpD) have not been completely elucidated in ICU patients. Generally, only *C. difficile* that produces toxin A (an intestinotoxin, *TcdA*) and/or toxin B (a cytotoxin, *TcdB*) can cause gastrointestinal diseases in humans. However, relationships between *C. difficile* type (i.e., A^+^B^+^, A^−^B^+^, and A^−^B^−^) and the gut microbiota composition have not been investigated.

In this prospective study, we utilized 16S rDNA and metagenome deep-sequencing to characterize the gut microbiota composition in healthy subjects, CDpD ICU patients, and *C. difficile* negative diarrhea (CDnD) ICU patients, as well as the gut microbiota composition in ICU patients with A^+^B^+^ CDpD, A^−^B^+^ CDpD, and A^−^B^−^ CDpD. Our results may help to elucidate the mechanisms underlying diarrhea and CDpD development in ICU patients and identify potential candidates for curative or preventive microbe therapy.

## Materials and Methods

### Study Population and Sample Collection

Xiangya Hospital is a 3,500-bed urban tertiary teaching hospital in Changsha, Hunan Province, China, and admits ~90,000 patients per annum. The hospital has a General ICU (35 beds). In this study, a total of 112 subjects were enrolled between March 2014 and December 2014. Subjects included 21 non-pregnant healthy individuals (to whom antibiotics had not been administered within 1 month and without any signs of diarrhea within 7 days before sample collection) and 91 non-pregnant ICU patients with signs of hospital-onset diarrhea (where diarrhea occurred 48 h after hospital admission with stool three or more times within 24 h). Subjects with inflammatory bowel disease and diarrhea that occurred less than 48 h after hospital admission were excluded from the study. For the ICU patients with hospital-onset diarrhea, *C. difficile* was screened within 24 h after sampling (at least 300-μl diarrheal stool samples frozen at−20°C for PCR) using 16S rDNA-PCR (PS13: 5′-GGAGGCAGCAGTGGGGAATA-3′, PS14: 5′-TGACGGGCGGTGTGTACAAG-3′) (Persson et al., 2011), sequencing, and BLAST alignment. Patients with positive results were classified into the CDpD group (*N* = 58), and patients with negative results were classified into the CDnD group (*N* = 33).

Diarrheal stool samples (at least 300 μl) obtained from patients and non-diarrheal stool samples (at least 300 μl) obtained from healthy individuals were frozen at −80°C before sequencing in 2015. Then, 16S rDNA V4 sequencing-based microbiota analysis was performed for stool samples in the CDpD, CDnD, and healthy control groups (CDpD-CDnD-control 16S rDNA V4 sequencing analysis). Moreover, 14 samples were selected from these groups (five samples in the CDpD group, four samples in the CDnD group, and five samples in the control group) to conduct genome sequencing-based metagenomics analysis (CDpD-CDnD-control metagenome sequencing analysis).

According to the PCR results of *C. difficile TcdA* (gene for toxin A; Forward primer: 5′-AGATTCCTATATTTACATGACAATAT-3′, Reverse primer: 5′-GTATCAGGCATAAAGTAATATACTTT-3′) (Lemee et al., 2004) and *TcdB* (gene for toxin B; NK104: 5′-GTGTAGCAATGAAAGTCCAAGTTTACGC-3′, NK105: 5′-CACTTAGCTCTTTGATTGCTGCACCT-3′) (Kato et al., 1998), patients in the CDpD group were classified into the A^+^B^+^ subgroup (*N* = 34), A^−^B^+^ subgroup (*N* = 7), and A^−^B^−^ subgroup (*N* = 17), and 16S rDNA V4 sequencing-based microbiota analysis was then performed for stool specimens in the A^+^B^+^, A^−^B^+^, and A^−^B^−^ subgroups (CDpD subgroups 16S rDNA V4 sequencing analysis).

### Clinical Data Collection

For each subject, clinical data were collected, including age, gender, duration of hospital stay, *C. difficile* detection, other diseases (i.e., diabetes, cancer, hematopathy, respiratory failure, renal insufficiency, and tuberculosis), surgery, and exposure to immunosuppressants, glucocorticoids, and antibiotics.

### Amplification and Sequencing of 16S rDNA

As previously described (Schubert et al., 2014; Milani et al., 2016), DNA was extracted from stool samples, and the V4-region (primers: 515F and 806R) of 16S rDNA were amplified and sequenced using an Illumina HiSeq2500 PE250 Paired-end sequencer. As detailed in the Supplemental Materials, 16S rDNA-based microbiota analysis was performed, including chimera sequence removal, operational taxonomic unit (OTU) clustering, taxonomic assignment, phylogenetic relationship, α-diversity analysis, and β-diversity analysis [unweighted unifrac distance, weighted unifrac distance, sample clustering tree, and principal coordinate analysis (PCoA)].

### Genome Sequencing

Total metagenomic DNA was isolated from each stool sample (Milani et al., 2016). After obtaining fragments of 300 bp (ultrasonic method), end repairing, adding poly-A, adding sequencing adapters, purification, and PCR, DNA libraries were obtained and then sequenced using an Illumina HiSeq2500 Paired-end sequencer with a 2 × 150 bp read length. Metagenomic data were analyzed (Supplemental Materials), including metagenome assembly *de novo*, gene calling, alignment of unigenes to reference genomes (microbial reference genomes of bacteria, fungi, archaea, and viruses in the NR database of the National Center for Biological Information), taxonomical analysis and pathway annotation.

### Statistical Analyses

Statistical analyses were performed using SPSS 19.0 software to determine the differences in clinical characteristics among groups (i.e., CDpD, CDnD, and control groups), as well as subgroups (i.e., A^+^B^+^, A^−^B^+^, and A^−^B^−^ subgroups). For continuous variables, comparison among three groups was performed using one-way ANOVA, whereas comparison between two groups was conducted using independent-samples t-test. In one-way ANOVA, least-significant-difference test and Dunnett's T3 method were used under the conditions of equal variance and unequal variance, respectively. *P* < 0.05 was set as the criterion for statistical difference. For categorical variables, Fisher's exact test and Pearson's chi-square test were performed.

## Results

### Clinical Features of Subjects

For CDpD-CDnD-control 16S rDNA V4 sequencing analysis, subjects in the CDpD group (*N* = 58), CDnD group (*N* = 33), and control group (*N* = 21) were significantly different for age, but similar for gender. Subjects in the CDpD and CDnD groups were significantly different for antibiotics usage (i.e., exposure to antibiotics within 1 month before diarrhea) but similar for the other clinical features (Table 1).

**Table 1:** Demographic and clinical characteristics of the population in each experimental group.

		**CDpD-CDnD-control 16S rDNA V4 sequencing analysis**				**CDpD subgroups 16S rDNA V4 sequencing analysis**				**CDpD-CDnD-control genome sequencing analysis**			
		**CDpD** **(*N* = 58)**	**CDnD** **(*N* = 33)**	**Control** **(*N* = 21)**	***P***	**A^**+**^B^**+**^** **(*N* = 34)**	**A^**−**^B^**+**^** **(*N* = 7)**	**A^**−**^B^**−**^** **(*N* = 17)**	***P***	**CDpD** **(*N* = 5)**	**CDnD** **(*N* = 4)**	**Control** **(*N* = 5)**	***P***
Age	Mean (SD)	55.3 (15.9)	58.8 (15.6)	36.6 (9.5)	<0.001	56.8 (17.3)	54.4 (10.1)	52.6 (15.3)	0.684	54.2 (21.4)	72.0 (11.7)	38.8 (13)	0.035
		CDpD vs. CDnD			0.276	A^+^B^+^ vs. A^−^B+			0.728	CDpD vs. CDnD			0.132
		CDpD vs. Control			<0.001	A^+^B^+^ vs. A^−^B^−^			0.392	CDpD vs. Control			0.164
		CDnD vs. Control			<0.001	A^−^B^+^ vs. A^−^B^−^			0.806	CDnD vs. Control			0.011
Gender	Female	14 (24.1%)	14 (42.4%)	8 (38.1%)	0.162	10 (29.4%)	0 (0%)	4 (23.5%)	0.253	3 (60%)	2 (50%)	0 (0%)	0.151
	Male	44 (75.9%)	19 (57.6%)	13 (61.9%)		24 (70.6%)	7 (100%)	13 (76.5%)		2 (40%)	2 (50%)	5 (100%)	
Inpatient days	Mean (SD)	29.4 (18.9)	27.2 (17.2)	NA	0.577	31.1 (20.4)	27.4 (11.4)	26.9 (18.6)	0.738	23.4 (19.8)	39.3 (7.7)	NA	0.157
						A^+^B^+^ vs. A^−^B^+^			0.649				
						A^+^B^+^ vs. A^−^B^−^			0.471				
						A^−^B^+^ vs. A^−^B^−^			0.955				
With diabetes	Yes	9 (15.5%)	2 (6.06%)	NA	0.183	6 (17.6%)	0 (0%)	3 (17.6%)	0.481	0 (0%)	0 (0%)	NA	NA
With cancer	Yes	6 (10.3%)	4 (12.1%)	NA	0.794	3 (8.82%)	1 (14.3%)	2 (11.8%)	0.844	0 (0%)	1 (25%)	NA	0.444
With hematopathy	Yes	2 (3.45%)	0 (0%)	NA	0.533	2 (5.88%)	0 (0%)	0 (0%)	0.650	0 (0%)	0 (0%)	NA	NA
With respiration failure	Yes	7 (12.1%)	4 (12.1%)	NA	0.994	4 (11.8%)	2 (28.6%)	1 (5.88%)	0.285	0 (0%)	1 (25%)	NA	0.444
With renal insufficiency	Yes	5 (8.62%)	3 (9.09%)	NA	0.939	4 (11.8%)	1 (14.3%)	0 (0%)	0.295	1 (20%)	1 (25%)	NA	1.00
With tuberculosis	Yes	0 (0%)	1 (3.03%)	NA	0.363	0 (0%)	0 (0%)	0 (0%)	NA	0 (0%)	0 (0%)	NA	NA
Antibiotics usage	Yes	39 (67.2%)	8 (24.2%)	NA	<0.001	29 (85.3%)	5 (71.4%)	5 (29.4%)	<0.001	5 (100%)	1 (25%)	NA	0.048
Immunosuppressant usage	Yes	0 (0%)	0 (0%)	NA	NA	0 (0%)	0 (0%)	0 (0%)	NA	0 (0%)	0 (0%)	NA	NA
Glucocorticoids usage	Yes	5 (8.62%)	0 (0%)	NA	0.083	3 (8.82%)	2 (28.6%)	0 (0%)	0.085	0 (0%)	0 (0%)	NA	NA
Surgery before diarrheal	Yes	27 (46.6%)	17 (51.5%)	NA	0.649	13 (38.2%)	4 (57.1%)	10 (58.8%)	0.318	5 (100%)	2 (50%)	NA	0.167

*CDpD, Clostridium difficile-positive diarrhea; CDnD, C. difficile-negative diarrhea; SD, standard deviation; NA, not applicable. Antibiotics usage, exposure to antibiotics within 1 month before diarrheal; Immunosuppressant usage, exposure to immunosuppressant within 1 month before diarrheal; Glucocorticoids usage, exposure to Glucocorticoids within 1 month before diarrheal; Surgery before diarrheal, acceptance of surgery before diarrheal*.

For the CDpD subgroups 16S rDNA V4 sequencing analysis, subjects in the A^+^B^+^ subgroup (*N* = 34), A^−^B^+^ subgroup (*N* = 7), and A^−^B^−^ subgroup (*N* = 17) of the CDpD group were significantly different for antibiotics usage but similar for the other clinical features (Table 1).

For CDpD-CDnD-control metagenome sequencing analysis, subjects in the CDpD group (*N* = 5), CDnD group (*N* = 4), and control group (*N* = 5) were significantly different for age but similar for gender. Subjects in the CDpD and CDnD groups were significantly different for antibiotics usage but similar for the other clinical features (Table 1).

### CDpD-CDnD-Control 16S rDNA V4 Sequencing Analysis

In α-diversity analysis, the Chao1 index, Shannon index, and number of observed species were assessed. The Chao1 index (i.e., community richness) in the CDpD group was lower than that in the CDnD and control groups. The Shannon index (i.e., community diversity) in the CDpD group was similar to that in the CDnD group but much lower than that in the control group. The number of observed species in the CDpD group was much lower than in the CDnD and control groups. A total of 2,125 16S rDNA-based OTUs were clustered, among which 513 OTUs were shared by the three groups.

**Figure 1:**
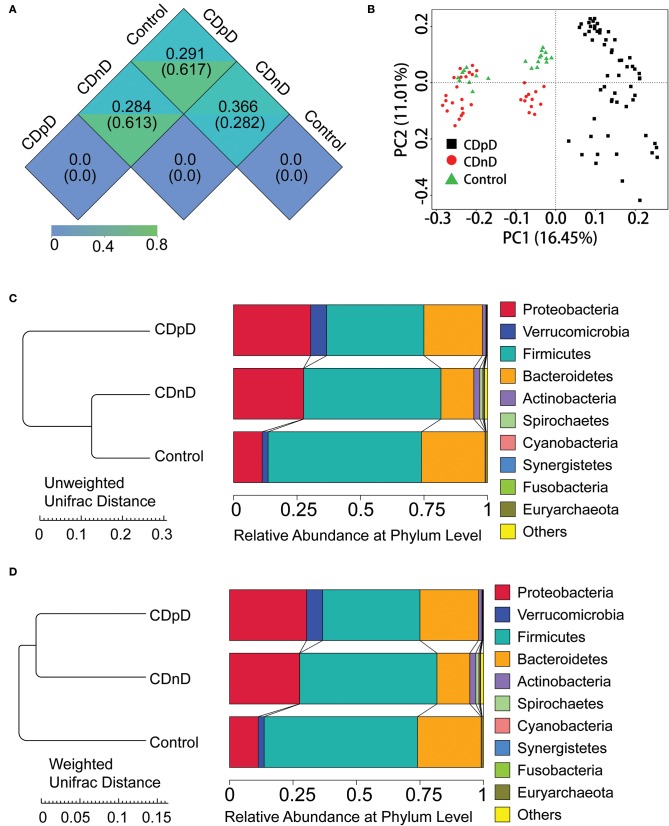
α-diversity and β-diversity analysis in CDpD-CDnD-control 16S rDNA V4 sequencing analysis. **(A)** Unifrac distance between groups. Upper number: weighted unifrac distance; lower number: un-weighted unifrac distance. Both numbers represent the index for differences in taxon-diversity between groups. **(B)** PCoA based on unweighted unifrac distance. **(C)** Sample clustering based on unweighted unifrac distance and relative abundance at Phylum level. **(D)** Sample clustering based on weighted unifrac distance and relative abundance at Phylum level.

In β-diversity analysis, the un-weighted unifrac distances were 0.282, 0.617, and 0.61 between CDnD and control, between CDpD and control, and between CDpD and CDnD, respectively (Figure 1A). PCoA was conducted based on un-weighted unifrac distance, and CDpD samples could be clearly separated from CDnD samples and controls (Figure 1B). Besides, the CDpD, CDnD, and control groups could be distinguished from each other based on unweighted/weighted unifrac distance and relative abundance at the Phylum level (Figures 1C,D).

**Figure 2:**
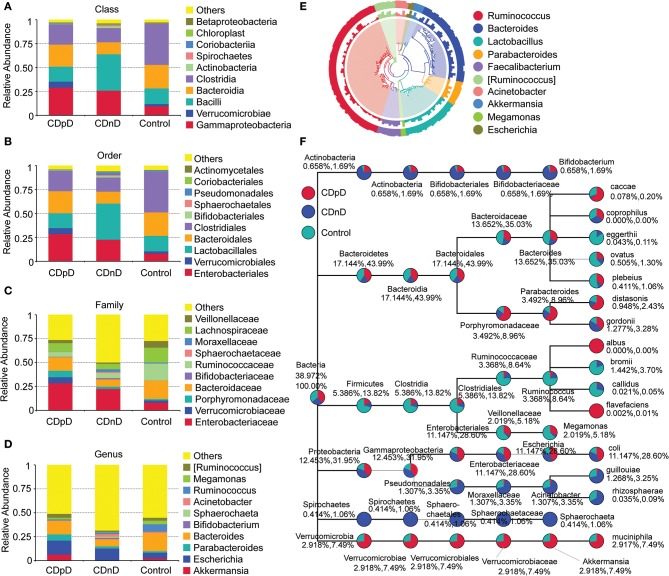
Taxonomic differences and phylogenetic relationships in CDpD-CDnD-control 16S rDNA V4 sequencing analysis. **(A)** Taxonomic differences at Class level. **(B)** Taxonomic differences at Order level. **(C)** Taxonomic differences at Family level. **(D)** Taxonomic differences at Genus level. **(E)** Phylogenetic relationships between OTUs annotated into top 10 Genera. Inner cycle, phylogenetic tree based on representative sequences of OTUs; middle cycle, relative abundances of OTUs; outer cycle, reliability of OUT annotation. **(F)** Taxonomic differences in selected taxa at the levels of Phylum, Class, Order, Family, and Genus. The former (later) number = [mean relative abundance of this taxon in all samples]/[mean relative abundance of all (selected) taxa in all samples] × 100%.

Relative abundances of the top 10 taxa in terms of Phylum, Class, Order, Family, and Genus were studied. At the Phylum level, the relative abundance of Firmicutes was lower whereas those of Proteobacteria and Verrucomicrobia were higher in the CDpD group when compared with the CDnD and control groups (Figures 1C,D). At the Class level, the relative abundances of Verrucomicrobiae and Gammaproteobacteria were higher in the CDpD group (Figure 2A). At the Order level, the relative abundances of Verrucomicrobiales and Enterobacteriales were higher in the CDpD group (Figure 2B). At the Family level, the relative abundances of Porphyromonadaceae, Verrucomicrobiaceae, and Enterobacteriaceae were higher in the CDpD group (Figure 2C). At the Genus level, the relative abundances of *Parabacteroides* and *Akkermansia* were higher in the CDpD group (Figure 2D).

The phylogenetic relationships between the representative sequences of all OTUs corresponding to the top 10 Genera are shown in Figure 2E. Additionally, a taxonomic tree was constructed (Figure 2F). The abundances of Porphyromonadaceae, *Parabacteroides, Parabacteroides distasonis*, and *Bacteroides caccae* in the CDpD group were greater than those in the CDnD and control groups. These taxa might be associated with CDpD. Firmicutes, Clostridia, Clostridiales, Ruminococcaceae, *Ruminococcus, Ruminococcus Bromii*, and *Ruminococcus callidus* as well as Veillonellaceae and *Megamonas* were more abundant in the control group than in the CDnD and CDpD groups. These taxa might be associated with diarrhea.

### CDpD-CDnD-Control Metagenome Sequencing Analysis

In core-pan gene analysis, the number of non-redundant genes declined along with the increase in sample number in the core-gene curve, whereas it increased along with the increase in sample number in the pan-gene curve. These results supported a further detailed analysis of metagenome sequencing data. A total of 591,552 genes were predicted based on metagenome sequences.

**Figure 3:**
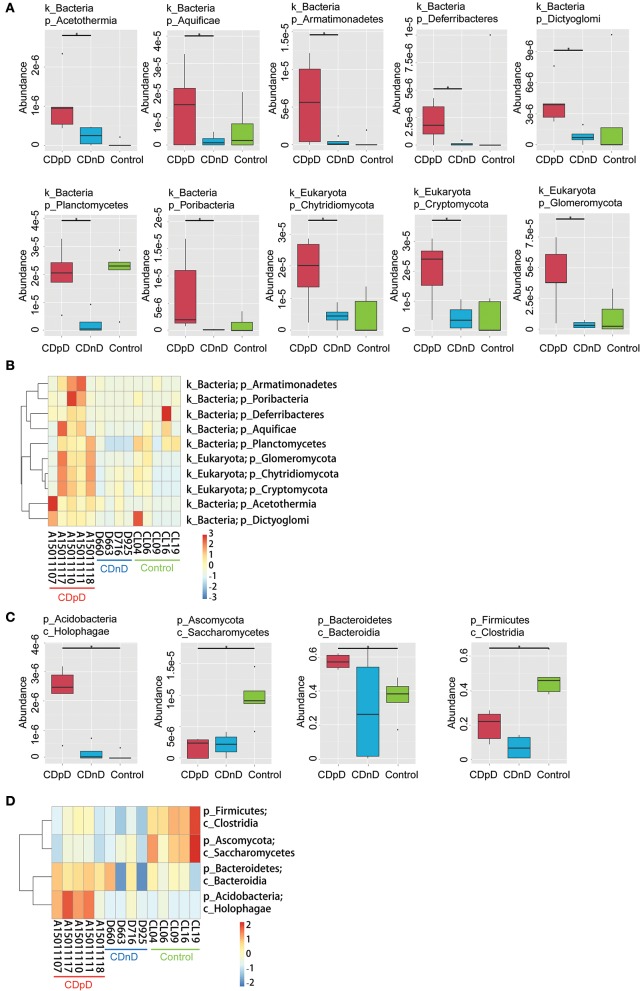
Significant taxonomic differences in CDpD-CDnD-control metagenome sequencing analysis. **(A)** *Significant taxonomic differences between groups (Phylum level). **(B)** Clustering based on the significant taxonomic differences at Phylum level. **(C)** *Significant taxonomic differences between groups (Class level). **(D)** Clustering based on the significant taxonomic differences at Class level.

In CDpD-CDnD-control metagenome sequencing analysis, significant differences were identified between the CDpD and CDnD groups in the abundances of 10 taxa (Dictyoglomi, Acetothermia, Cryptomycota, Chytridiomycota, Glomeromycota, Planctomycetes, Aquificae, Deferribacteres, Poribacteria, and Armatimonadetes; Figures 3A,B). Also, significant differences were found between the CDpD and control groups in the abundances of Holophagae, Bacteroidia, Saccharomycetes, and Clostridia (Figures 3C,D). In both CDpD-CDnD-control 16S rDNA V4 sequencing analysis and CDpD-CDnD-control metagenome sequencing analysis, the relative abundance of Clostridia in the CDpD group was lower than in the control group.

**Figure 4:**
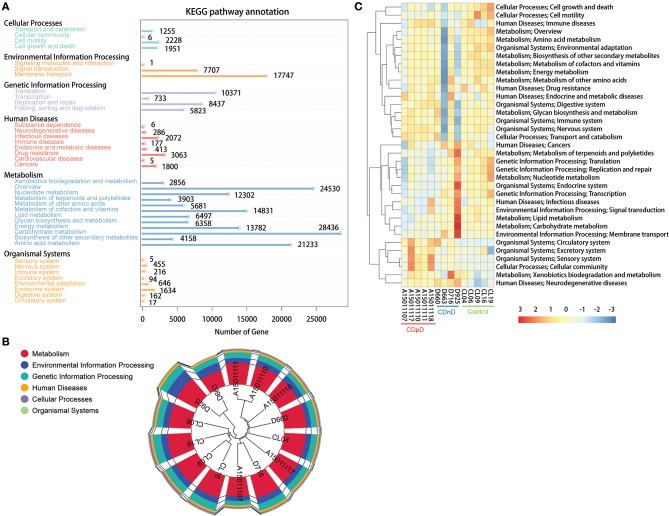
Functional annotation based on the KEGG database in CDpD-CDnD-control metagenome sequencing analysis. **(A)** Distribution of genes in KEGG pathways. **(B)** Sample clustering based on Bray-Curtis distances and gene abundances (Level 1). **(C)** Top-35 pathways (Level 2) with highest gene abundance.

Based on the Kyoto Encyclopedia of Genes and Genomes (KEGG) database, most of the predicted genes were related with Metabolism (e.g., Carbohydrate metabolism, Amino acid metabolism), Environmental Information Processing (e.g., Membrane transport), and Genetic Information Processing (e.g., Translation) (Figure 4A). Samples were clustered based on Bray-Curtis distances and gene abundances (KEGG level 1), and control samples were closely clustered (Figure 4B). The top-35 pathways with the highest gene abundance were identified (KEGG level 2, Figure 4C). Compared with CDnD and control samples, CDpD samples showed significantly higher abundances in pathways such as “Cellular community,” “Excretory system,” and “Circulatory system.” Besides, “Lysine biosynthesis” and “Coenzyme B biosynthesis” were only annotated in CDpD samples when compared with controls.

### CDpD Subgroups 16S rDNA V4 Sequencing Analysis

In α-diversity analysis, the Chao1 index, Shannon index, and number of observed species in the A^+^B^+^ subgroup was much higher than in the A^−^B^+^ and A^−^B^−^ subgroups.

**Figure 5:**
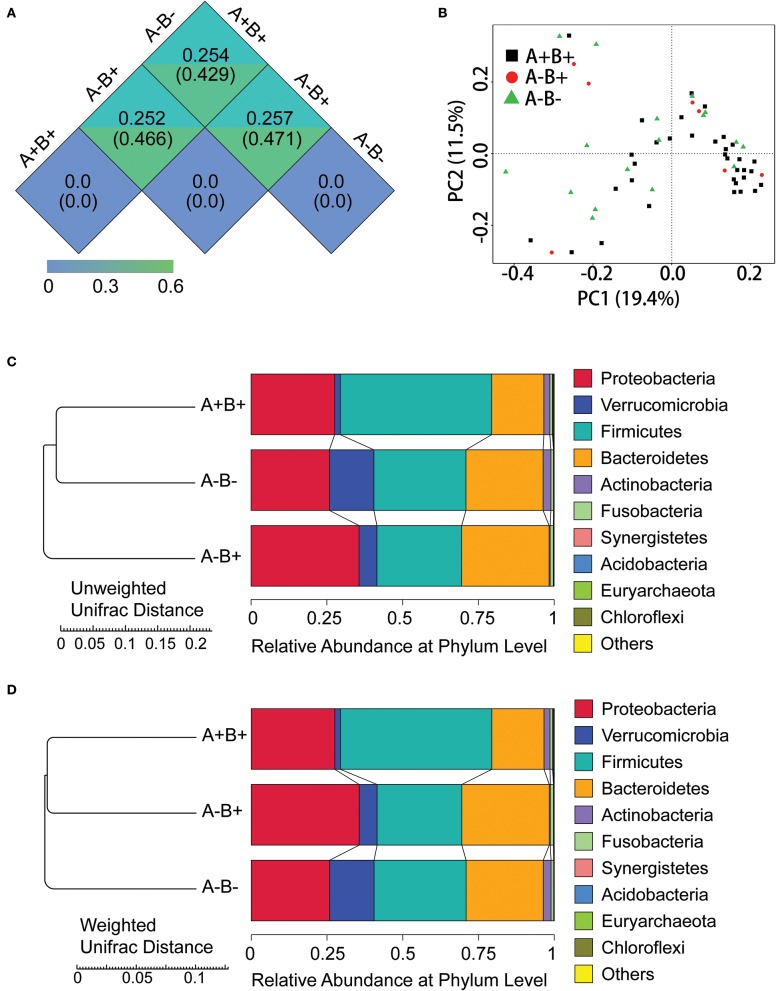
α-diversity and β-diversity analysis in subgroups 16S rDNA V4 sequencing analysis. **(A)** Unifrac distance between groups. Upper number: weighted unifrac distance; lower number: un-weighted unifrac distance. Both numbers represent the index for differences in taxon-diversity between groups. **(B)** PCoA based on unweighted unifrac distance. **(C)** Sample clustering based on unweighted unifrac distance and relative abundance at Phylum level. **(D)** Sample clustering based on weighted unifrac distance and relative abundance at Phylum level.

In β-diversity analysis, the unweighted unifrac distances were 0.471, 0.429, and 0.466 between A^−^B^+^ and A^−^B^−^, between A^+^B^+^ and A^−^B^−^, and between A^+^B^+^ and A^−^B^+^ (Figure 5A). In β-diversity analysis, the weighted unifrac distances were 0.257, 0.254, and 0.252 between A^−^B^+^ and A^−^B^−^, between A^+^B^+^ and A^−^B^−^, and between A^+^B^+^ and A^−^B^+^ (Figure 5A). These results indicated that the differences in taxon diversity between these subgroups were similar.

In PCoA based on unweighted unifrac distance, high inter-sample variability was found across groups, and A^+^B^+^ samples could not be distinguished from A^−^B^+^ or A^−^B^−^ samples (Figure 5B). However, the A^+^B^+^, A^−^B^+^, and A^−^B^−^ subgroups could be distinguished from each other based on the unweighted/weighted unifrac distance and relative abundance at the Phylum level (Figures 5C,D).

**Figure 6:**
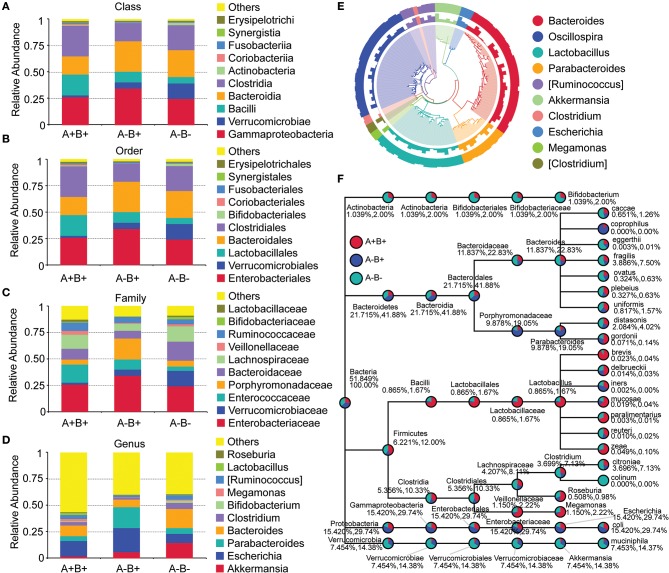
Taxonomic differences and phylogenetic relationships in subgroups 16S rDNA V4 sequencing analysis. **(A)** Taxonomic differences between A^+^B^+^, A^−^B^+^, and A^−^B^−^ subgroups (Class level). **(B)** Taxonomic differences between A^+^B^+^, A^−^B^+^, and A^−^B^−^ subgroups (Order level). **(C)** Taxonomic differences between A^+^B^+^, A^−^B^+^, and A^−^B^−^ subgroups (Family level). **(D)** Taxonomic differences between A^+^B^+^, A^−^B^+^, and A^−^B^−^ subgroups (Genus level). **(E)** Phylogenetic relationships between OTUs annotated into top 10 Genera. Inner cycle, phylogenetic tree based on representative sequences of OTUs; middle cycle, relative abundances of OTUs; outer cycle, reliability of OUT annotation. **(F)** Taxonomic differences in selected taxa at the levels of Phylum, Class, Order, Family, and Genus. The former number = (mean relative abundance of this taxon in all samples)/(mean relative abundance of all taxa in all samples) × 100%; the later number = (mean relative abundance of this taxon in all samples)/(mean relative abundance of selected taxa in all samples) × 100%.

At the Phylum level, the relative abundances of Bacteroidetes and Verrucomicrobia were lower whereas the relative abundance of Firmicutes was higher in the A^+^B^+^ subgroup when compared with the A^−^B^+^ and A^−^B^−^ subgroups (Figures 5C,D). At the Class level, the relative abundances of Bacteroidia and Verrucomicrobiae were lower and the relative abundances of Clostridia and Bacilli were higher in the A^+^B^+^ subgroup (Figure 6A). At the Order level, the relative abundances of Bacteroidales and Verrucomicrobiales were lower and the relative abundances of Clostridiales and Lactobacillales were higher in the A^+^B^+^ subgroup (Figure 6B). At the Family level, the relative abundance of Verrucomicrobiaceae was lower and the relative abundances of Enterococcaceae and Ruminococcaceae were higher in the A^+^B^+^ subgroup (Figure 6C). At the Genus level, the relative abundance of *Akkermansia* was lower in the A^+^B^+^ subgroup (Figure 6D). Moreover, Methylococcaceae, Geobacteraceae, Peptococcaceae, Crenotrichaceae, Mogibacteriaceae, *Acetobacter, Dialister*, and *Crenothrix* significantly increased in the A^+^B^+^ subgroup in comparison with the A^−^B^−^ subgroup. Exiguobacteraceae, Rickettsiaceae, Promicromonosporaceae, Procabacteriaceae, *Providencia, Cellulosimicrobium, Wolbachia*, and *Saccharopolyspora* significantly decreased in the A^+^B^+^ subgroup in comparison with the A^−^B^−^ subgroup.

Phylogenetic relationships between the representative sequences of all OTUs corresponding to the top 10 taxa in Genus were studied (Figure 6E), and a taxonomic tree was constructed (Figure 6F). *Bacteroides eggerthii, Bacteroides fragilis, Bacteroides ovatus*, and *Clostridium citroniae* were more abundant in the A^−^B^−^ subgroup than in the A^−^B^+^ and A^+^B^+^ subgroups. These taxa might be suppressed by the presence of toxins. The abundances of Proteobacteria, Gammaproteobacteria, Enterobacteriales, Enterobacteriaceae, *Escherichia*, and *Escherichia coli* in the A^−^B^−^ subgroup A^−^B^+^ subgroup were much lower than in the A^+^B^+^ and A^−^B^−^ subgroups. These taxa might be associated with A^−^B^+^.

## Discussion

To better understand how the microbiome is associated with diarrhea, CDpD, and *C. difficile* type, we characterized the gut microbiota of healthy individuals and ICU individuals with CDpD or CDnD. Consequently, we found gut microbiota compositions with potential associations with CDpD and *C. difficile* type in ICU patients.

Analyses of the Shannon index and number of observed species indicated that the microbial diversity of the CDpD group was lower than that of the CDnD and control groups, and this agreed well with a previous study in elderly (age ≥ 65) hospitalized patients (Milani et al., 2016). The unweighted unifrac distance between CDpD and control/CDnD was higher than that between CDnD and control. In PCoA, CDpD samples could be distinguished from CDnD samples and controls, whereas CDnD samples could not be separated from controls. These results indicated that the differences in taxon diversity between CDpD and control/CDnD were much higher than those between CDnD and control and that the appearance of *C. difficile* was strongly associated with the decrease in diversity of the gut microbiota.

Differences in community composition between groups were investigated. Metagenome analysis revealed that the abundances of 10 taxa (e.g., Deferribacteres, Cryptomycota, Acetothermia) in the CDpD group were significantly higher than in the CDnD group. Additionally, the abundances of two taxa (Saccharomycetes and Clostridia) were significantly lower in CDpD in comparison with control. *Saccharomyces boulardii* is a nonpathogenic yeast that promotes intestinal immunoglobulin-A immune response to *C. difficile* toxin A and thus protects against recurrent *C. difficile* colitis and diarrhea (Qamar et al., 2001). A balanced gut microbiota is characterized by conserved features like the predominance of Bacteroidia and Clostridia (Winter and Baumler, 2014). Therefore, CDpD might be associated with a decrease in beneficial taxa (i.e., Saccharomycetes and Clostridia) and an increase in harmful taxa (e.g., Deferribacteres, Cryptomycota, Acetothermia) in gut microbiota. Also, Saccharomycetes and Clostridia might serve as future candidate targets in ICU CDpD patients to help them re-establish healthy gut microbiota. *Saccharomyces boulardii* has been utilized to prevent antibiotic-associated diarrhea in adult hospitalized patients in clinical trials (Ehrhardt et al., 2016).

Moreover, based on 16S rDNA sequencing data, relative abundances of some taxa (e.g., Gammaproteobacteria, Enterobacteriaceae, Porphyromonadaceae, and *Akkermansia*) were higher in the CDpD group in comparison with the CDnD and control groups. Our results were partially consistent with previous study, in which Gammaproteobacteria and Enterobacteriaceae were significantly over-represented in CDI samples, whereas Porphyromonadaceae and *Akkermansia* were significantly under-represented in CDI samples, in comparison with non-CDI samples. Reportedly, *A. muciniphila* is an intestinal representative of the Verrucomicrobia, and it associates with health in humans. Reduction of *A. muciniphila* was observed in patients with inflammatory bowel diseases, which led to the hypothesis that *A. muciniphila* possesses health-promoting activities and anti-inflammatory properties (Derrien et al., 2016). Although *Akkermansia* may improve barrier function, its over-representation in CDpD may be caused by modifications in the gut micro-environment and reflects enteric mucosa inflammation (Zwielehner et al., 2011). Thus, CDpD might also correlate with increase in *Akkermansia*.

Furthermore, differences in microbiota pathways between the three groups were studied using metagenome sequencing data. “Lysine biosynthesis” and “Coenzyme B biosynthesis” were only annotated in CDpD samples. Therefore, CDpD might be related with these pathways in gut microbiota, and drugs inhibiting these pathways or the colonization of Deferribacteres, Cryptomycota, Acetothermia, Gammaproteobacteria, and Enterobacteriaceae might be effective in limiting *C. difficile* blooming.

In the CDpD group, the community richness and microbial diversity of the A^+^B^+^ subgroup were higher than those of the A^−^B^+^ and A^−^B^−^ subgroups. The similar unweighted unifrac distances (or weighted unifrac distances) between A^−^B^+^ and A^−^B^−^, between A^+^B^+^ and A^−^B^−^, and between A^+^B^+^ and A^−^B^+^ indicated similar differences in taxon diversity between these subgroups. High inter-sample variability was found in PCoA, and A^+^B^+^ samples could not be distinguished from A^−^B^+^ or A^−^B^−^ samples. Besides, some taxa were significantly different between the A^+^B^+^ and A^−^B^−^ subgroups (e.g., *Acetobacter, Saccharopolyspora*). These results suggested that higher sample numbers in the A^+^B^+^, A^−^B^+^, and A^−^B^−^ subgroups are required for conclusive results.

Unfortunately, some limitations were found in this study. Firstly, we did not collect the reasons for diarrhea in the CDnD group. Secondly, age and antibiotic usage were not matched among the three groups, as the clinical complexity of ICU patients and the application of polypharmacy did not allow us to identify a sufficient number of subjects for each group or to standardize antibiotic treatment. In our future studies, more subjects will be enrolled to obtain a better stratification of patient features at baseline, and more clinical data (e.g., the reasons for diarrhea in the CDnD group) will be collected.

## Conclusions

In ICU patients, the development of CDpD might be associated with alterations in gut microbiota, including decreases in beneficial taxa (i.e., Saccharomycetes and Clostridia) and increases in Deferribacteres, Cryptomycota, Acetothermia, Gammaproteobacteria, and Enterobacteriaceae. Besides, *C. difficile* toxin types might slightly influence gut microbiota composition in ICU patients with CDpD. These results provided a further understanding of the mechanism of CDpD in ICU patients and shed light on future directions for developing novel therapies to re-construct healthy gut microbiota.

## Data Availability Statement

The data supporting the conclusion of this article were submitted to SRA NCBI; the SRA accession information is PRJNA597086 (16s rDNA) and PRJNA591064 (metagenome).

## Ethics Statement

This study was approved by the Ethics Committee of Xiangya Hospital Central South University, China (ID 201212027). All participants granted written informed consent before enrollment, and all investigations followed the principles of the Declaration of Helsinki.

## Author Contributions

AW, JD, and CL conceived and designed the experiments. JD, SL, CZ, and XM performed the experiments. CF, PZ, QD, and CL analyzed the data. CL and AW wrote the paper. All authors read and approved the final manuscript.

## Conflict of Interest

The authors declare that the research was conducted in the absence of any commercial or financial relationships that could be construed as a potential conflict of interest.

## Abbreviations

CDI: *C. difficile* infection
CDpD: *C. difficile*-positive diarrhea
FMT: Fecal microbiota transplantation
ICU: Intensive care unit
OTU: Operational taxonomic unit.
