# Supplementary material for: Gut Microbiota Composition Associated With Clostridium difficile-Positive Diarrhea and C. difficile Type in ICU Patients
Source: Front Cell Infect Microbiol. 2020 May 11;10:190. doi: 10.3389/fcimb.2020.00190 (PMC7233261; doi:10.3389/fcimb.2020.00190)
Supplement: Supplementary file 1 [file Data_Sheet_1.docx]

**16S rDNA-based microbiota analysis**

***Operational Taxonomic Unit (OTU) clustering***

After data preprocessing and removing chimera sequences, UPARSE (version: 7.0.1001, <http://drive5.com/uparse/>) [^1^](#_ENREF_1) was used to cluster effective tags into different OTUs (identity ≥ 97%). The tag with highest frequency in a certain OTU was considered as the representative sequence of this OTU.

***Taxonomic assignment***

Taxonomic assignments were performed using the Ribosomal Database Project classifier (version: 2.2, <http://sourceforge.net/projects/rdp-classifier/>) [^2^](#_ENREF_2) and GreenGene database (<http://greengenes.lbl.gov/cgi-bin/nph-index.cgi>) [^3^](#_ENREF_3) with a confidence threshold of 0.8-1. Community composition was analyzed at the levels of Kingdom, Phylum, Class, Order, Family, Genus and Species, respectively.

***Phylogenetic relationship***

MUSCLE (version: 3.8.31, <http://www.drive5.com/muscle/>) [^4^](#_ENREF_4) was utilized to investigate the phylogenetic relationships between the representative sequences of OTUs via conducting multi-sequence alignment.

***Analysis of α-diversity***

Normalization was performed between all samples based on the minimum sequencing amount in the samples. Then, QIIME (version: 1.7.0) was utilized to analyze α-diversity. Community richness and diversity within community were assessed using rarefaction-curve, Chao estimator (<http://scikit-bio.org/docs/latest/generated/generated/skbio.diversity.alpha.chao1.html#skbio.diversity.alpha.chao1>, a community richness indicator) and Shannon index (<http://scikit-bio.org/docs/latest/generated/generated/skbio.diversity.alpha.shannon.html#skbio.diversity.alpha.shannon>, a community diversity indicator). Results were visualized using R software (version: 2.15.3).

***Analysis of β-diversity***

QIIME was utilized to analyze β-diversity (i.e. between-habitat diversity). Differences of community composition between samples were assessed using un-weighted unifrac distance, weighted unifrac distance, sample clustering tree, principal component analysis (PCA; tool: FactoMineR package and ggplot2 package in R software) and principal co-ordinates analysis (PCoA; tool: WGCNA, stats, and ggplot2 packages in R software).

**Analysis of metagenomic data**

***Metagenome assembly***

After data preprocessing, clean reads with high-quality were *de novo* assembled into scaffolds using SOAPdenovo software (version: 2.21, <http://soap.genomics.org.cn/soapdenovo.html>) [^5^](#_ENREF_5) with parameters of -d 1, -M 3, -R, -u and -F (k-mer length was set as 49, 55 or 59, and only the k-mer with maximum N50 was finally used). Scaffolds were broken into scaftigs via deleting N bases. Then, SoapAligner software was used to align clean reads with these scaftigs (alignment parameters: -u, -2, -m 200), and the reads which did not match these scaftigs were extracted from every sample and pooled together to identify information of rare species. Pooled-reads were also assembled into scaffolds (k-mer length = 55), which were broken into scaftigs. Scaftigs from each sample and pooled-reads were filtered, and scaftigs with a length of ≥ 500 bp were used for statistics and gene calling.

***Gene calling***

Open reading frames (ORFs) were predicted using scaftigs (≥500 bp) and MetaGeneMark software (version: 2.10, <http://exon.gatech.edu/GeneMark/meta_gmhmmp.cgi>) [^6^](#_ENREF_6), and ORFs with length <100 nt were removed. Then, a non-redundant gene catalogue was obtained using CD-HIT software (version: 4.5.8, <http://www.bioinformatics.org/cd-hit/>, parameters: sequence identity ≥0.95, coverage ≥0.9, -c 0.95, -G 0, -aS 0.9, -g 1, -d 0) [^7^](#_ENREF_7). Thereafter, SoapAligner software was utilized to align clean reads with this gene catalogue (alignment parameters: -m 200, -x 400, identity ≥ 95%), and genes matched with >2 reads were defined as unigenes. Abundance of each gene in each sample was assessed based on the number of matched reads (r) and gene length (L):

$$G_{k}=\frac{r_{k}}{L_{k}}\div\sum_{i=1}^{n} \frac{r_{k}}{L_{k}}$$

Gene abundance was used to perform core-pan gene analysis, sample correlation analysis and Venn analysis of gene count.

***Alignment of unigenes to reference genomes and taxonomical analysis***

DIAMOND software [^8^](#_ENREF_8) was used to align the metagenomic unigenes to microbial reference genomes of bacteria, fungi, archaea and viruses in the NR database (version: 2014-10-19) of the National Center for Biological Information (parameters: e-value for BLASTp ≤ 1×10^-5^). For each unigene, only the mapping results with e-value ≤ 10 × minimum e-value were remained, and LCA algorithm in MEGAN software [^9^](#_ENREF_9) was utilized to identify the minimum taxonomic rank without dissent caused by different mapping results.

The abundance of a certain taxon (kingdom, phylum, class, order, family, genus, or species) in a sample was defined as a sum of the abundances of all genes on its member genomes in the sample. The abundances of taxa in every sample were utilized to conduct Krnoa analysis, top-taxa display, sample-taxon clustering, PCA, sample clustering based on Bray-Curtis distances among gene abundances, taxon difference analysis (tool: Metastats [^10^](#_ENREF_10)), etc.

***Pathway annotation***

To annotate genes, DIAMOND [^8^](#_ENREF_8) was used to align unigenes with Kyoto Encyclopedia of Genes and Genomes (KEGG) database [^11^](#_ENREF_11) (parameters: e-value for BLASTp ≤ 1×10^-5^), and results with one HSP > 60 bits were remained.

The counts of annotated genes and the abundances of functions in every sample were utilized to conduct sample-pathway clustering, top-functions display, PCA based on function abundances, sample clustering based on Bray-Curtis distances among function abundances, function difference analysis (tool: Metastats [^10^](#_ENREF_10)), KEGG pathway display, etc.

**References**

(1) Edgar RC. UPARSE: highly accurate OTU sequences from microbial amplicon reads. Nat Methods. 2013;10:996-8.

(2) Wang Q, Garrity GM, Tiedje JM, Cole JR. Naive Bayesian classifier for rapid assignment of rRNA sequences into the new bacterial taxonomy. Applied and environmental microbiology. 2007;73:5261-7.

(3) DeSantis TZ, Hugenholtz P, Larsen N, et al. Greengenes, a chimera-checked 16S rRNA gene database and workbench compatible with ARB. Applied and environmental microbiology. 2006;72:5069-72.

(4) Edgar RC. MUSCLE: multiple sequence alignment with high accuracy and high throughput. Nucleic acids research. 2004;32:1792-7.

(5) Luo R, Liu B, Xie Y, et al. SOAPdenovo2: an empirically improved memory-efficient short-read de novo assembler. GigaScience. 2012;1:18.

(6) W. Z. Improvement of ab initio methods of gene prediction in genomic and metagenomic sequences [Dissertation]: Georgia Institute of Technology; 2010.

(7) Li W, Godzik A. Cd-hit: a fast program for clustering and comparing large sets of protein or nucleotide sequences. Bioinformatics (Oxford, England). 2006;22:1658-9.

(8) Buchfink B, Xie C, Huson DH. Fast and sensitive protein alignment using DIAMOND. Nat Methods. 2015;12:59-60.

(9) Huson DH, Mitra S, Ruscheweyh HJ, Weber N, Schuster SC. Integrative analysis of environmental sequences using MEGAN4. Genome research. 2011;21:1552-60.

(10) Paulson JN, Pop M, Bravo HC. Metastats: an improved statistical method for analysis of metagenomic data. Genome Biol. 2011;12:P17.

(11) Kanehisa M, Goto S, Sato Y, Kawashima M, Furumichi M, Tanabe M. Data, information, knowledge and principle: back to metabolism in KEGG. Nucleic acids research. 2014;42:D199-205.
